# Disposable Nitrile Glove Resistance to Limonene: Dextrous Robot Hand Versus ASTM F739 Comparison

**DOI:** 10.1021/acs.chas.3c00117

**Published:** 2024-03-20

**Authors:** Sean Banaee, Airek Mathews, Shane Que Hee

**Affiliations:** Department of Environmental Health Sciences and UCLA Center for Occupational & Environmental Health, Fielding School of Public Health, University of California Los Angeles, 650 Charles E Young Jr Drive South, Los Angeles, California 90095-1772, United States

**Keywords:** limonene, whole glove permeation, dextrous robot hand, glove thickness, ASTM F739, multivariate analysis

## Abstract

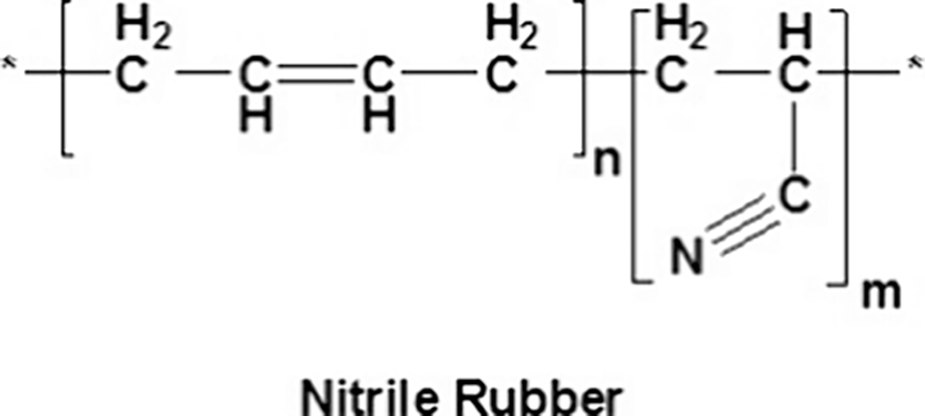

The current technique to assess glove resistance to chemicals for worker protection relies on challenging a flat, 2.54 cm diameter glove piece at or near room temperature. This does not simulate a donned whole glove near the skin temperature subjected to work activity forces. Four different types of disposable nonpowdered unlined/unsupported nitrile gloves in triplicate were measured for thickness, porosity, and for the acrylonitrile content (*A*) of the challenge and collection sides. Limonene permeation at 35 °C through a whole glove on a clenching and nonclenching dextrous robot hand and with the standard ASTM F739 technique were facilitated by taking samples from the collection sides for GC-MS analysis. The standardized breakthrough time (SBT) when permeation reached 100 ng/cm^2^/min and the steady state permeation rate (SSPR) depended on *A*, thickness, and porosity. Only the thinnest glove (Lavender) showed statistically significant (*p* ≤ 0.05) increased average SSPR for the clenching hand relative to the nonclenching hand and for the ASTM technique. The ASTM test data for the three thickest gloves were not statistically different from those of the robot hand, but differed from the manufacturer’s. More research with different chemicals and higher clenching forces is needed. Clenching forces can enhance the permeation. Workers wearing ultrathin disposable nitrile gloves have a higher potential for chemical penetration/permeation. Company glove permeation data obtained near room temperature may have a longer SBT and lower SSPR than in practice. Double gloving may be advisible in emergencies and for unknown chemicals when no appropriate thicker Chemical Protective glove is available.

## 1 Introduction

In 2019 before the COVID-19 pandemic, skin exposure caused 18,200 recordable illnesses across all industries in the United States, about 1.7 times those for respiratory diseases.^1^ A 2017 report also stated that skin diseases comprised 20–30% of occupational diseases in Europe.^2^ Protection of the skin of the hands to prevent such illnesses from chemical exposure occurs usually by wearing the “appropriate gloves”.

The major glove resistance tests for chemical permeation involve continuous chemical contact with small circular pieces of flat glove material as published by the American Society for Testing and Materials International (ASTM) F739,^3^ the International Standards Organization (ISO) 6529, or the European Community (EN) 374, the latter two combining in 2016.^4^ These tests are generally performed near room temperatures 20–27 °C by glove providers, with 27 °C being specified by the 2020 revision of ASTM F739. The thicker gloves worn in industry are part of the Chemical Protective Clothing (CPC). The thinner disposable gloves that allow facile manipulation of workpieces and small objects are also much worn in industry and predominate for laboratory and healthcare workers and for lay people to keep their hands dry. Disposable gloves may also be the only ones available when emergencies occur. Tight-fitting disposable gloves do quickly equilibrate to the average skin temperature of 35 °C as acknowledged in ASTM D-6978 for disposable gloves to protect against antineoplastic chemicals in aqueous solutions relative to healthcare worker protection.^5^ CPC gloves, when worn over a shift, also equilibrate to skin temperature. The permeation standards test an immobilized part of the glove (palm or top of palm) rather than the whole glove that experiences forces when donned, worn, and doffed that are not simulated in the standards. Two reviews have discussed these aspects at length.^6,7^

Whole glove permeation testing has been advocated^6−12^ as a better simulation of the permeation of a donned glove than a circular piece of material. The utilization of a dextrous robot hand that clenches^8,10−12^ has allowed simulation of the role of forces on the whole glove permeation of chemicals. This previous work showed that the time when cyclohexanol permeation at 35 °C reached 100 ng/cm^2^/min (standardized breakthrough time SBT) and the steady state permeation rate (SSPR) magnitude depended on disposable nitrile glove thickness (*L*) when a 1.8 kg clenching force was applied by the Yaeger dextrous robot hand (YDRH).^10,11^ An anthropometric dextrous robot hand is the nearest *in vitro* approach to an actual clenching human hand.^13^

The present investigation explores the relationships for SBT and SSPR with *L*, acrylonitrile content (*A*), and porosity (*P*) for the d-limonene and nitrile disposable whole glove interaction for the YDRH relative to data from the ASTM F739–12 methodology^3^ featuring a permeation cell of 1 in. (2.54 cm) diameter in a moving tray water bath, all permeation systems being at 35 °C. d-Limonene (1-methyl-4-prop-1-en-2-ylcyclohexene; CAS RN 5989–27–5; molecular weight 136.23 g/mol; boiling point 178 °C; water solubility 13.8 mg/L at 25 °C; vapor pressure 1.98 mmHg at 25 °C) was chosen as the challenge solvent because of its many uses where a citrus odor and flavor were needed in flavorings, fragrances, cosmetics, repellants of animals and insects, and when used as a pesticide.^14^

## 2 Materials and Methods

### 2.1 Materials

The nitrile disposable gloves were Kimberly Clark Professional Kimtech Science Blue, Purple, Sterling, and Lavender, unsupported, unlined, and powderless, from Fisher Scientific, Pittsburgh PA. d-Limonene (96%; hereafter called limonene) and 4-bromophenol (97%) internal standard (IS) were from Acros Organics, ThermoFisher, Grand Island, NY. The unassembled YDRH was purchased from Scientifics Direct, Tonawanda, NY.

### 2.2 Robot Hand System

**Figure 1:**
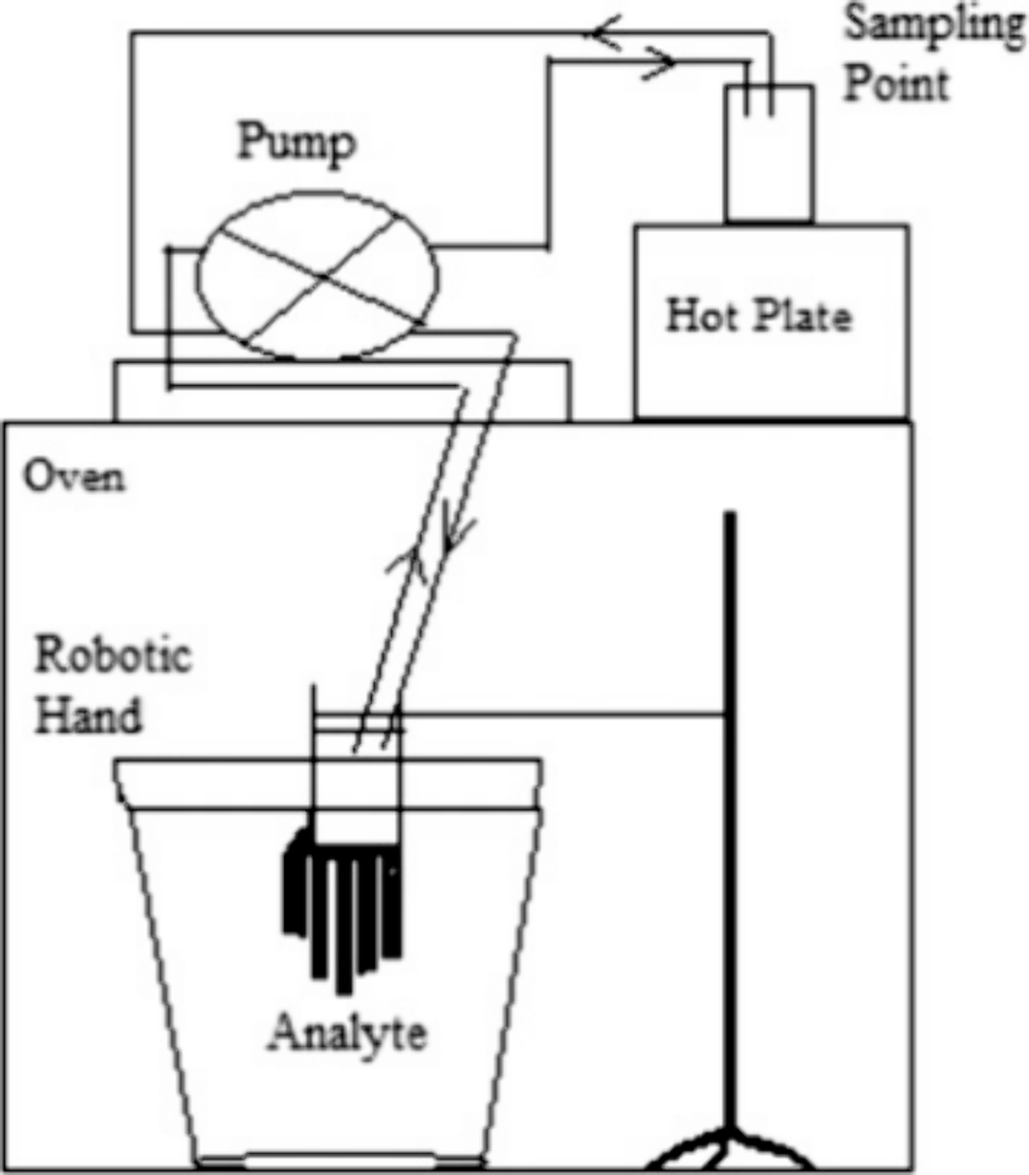
Whole glove dextrous robot hand permeation schematic diagram reprinted with permission under a cc-by-nc-sa creative commons license from order number 5744390668289. Copyright 2017/Oxford University Press.

The assembled YDRH (Figure 1) was part of a closed-loop dynamic flow system that recirculated collection Milli-Q water solvent by a peristaltic pump (Reglo 2-channel Var-Speed Analog pump from Cole Parmer, Court Vernon Hills, IL) at 100 mL/min between the inside surface of the test disposable nitrile glove and the outside surface of the CPC Solvex powderless unsupported/unlined nitrile glove (Ansell, Iselin, New Jersey) that protected the YDRH.^10,11^ The inlet and outlet of the YDRH were connected to the sampling point by Viton tubing (2.79 mm inner diameter extension and three-stop, Cole Parmer, Vernon Hill, IL). The hand in exposure situations was immersed inverted, held by the wrist from a clamp, in limonene contained in a vacuum desiccator (5-L from Fisher Scientific) within a Precision Econotherm Laboratory Oven (Fisher Scientific) at 35 °C (Figure 1). The hand was powered as described elsewhere to produce a 1.8 kg clenching force.^8,10,11^ Thicknesses of the conditioned and reconditioned gloves after each permeation experiment were measured with an Electronic Digital Micrometer Model CO-030025 (0–25 mm, 0.001 mm resolution) from Fisher Scientific. Permeation experiments were performed in triplicate.

### 2.3 ASTM F739–12 Modified Method

Four ASTM F739–12 2.54 cm leak-free permeation cells (I-PTC 600, Pesce Laboratories, Kennett Square PA) that were closed-loop collection without recirculation and containing 2.54 cm diameter exposure area of the palm part of the glove conditioned at room temperature at 52% relative humidity were placed in a moving tray water bath (Fisher Scientific Model 125 No. 429) at 35 °C as described elsewhere.^10,11^ A collection volume of 10 mL of Milli-Q deionized and organic-free water was pipetted into each permeation cell followed by a challenge limonene volume of 10 mL for three of the cells. Thicknesses of the conditioned and reconditioned glove pieces after each permeation experiment were measured as shown in Section 2.2.

### 2.4 Sample Collection

Shaking (ASTM) or YDRH clenching were stopped to take 100 μL (ASTM) or 1.0 mL samples (YDRH) for analysis by gas chromatography–mass spectrometry (GC-MS) at time intervals of 1, 10, 20, 40, 60, 80, 120, 240, 360, and 480 min. They were frozen after sampling.

### 2.5 Gas Chromatography–Mass Spectrometry (GC-MS)

Capillary GC-MS analyses by the internal standard method were performed on an Agilent 6890N gas chromatograph with a nonpolar HP-5MS fused silica capillary column, 60 m length × 0.42 mm external diameter, 0.32 mm internal diameter, 1 μm internal film thickness operated in the splitless mode in tandem with an Agilent 5973 Mass Selective Detector (MSD) (Agilent Technologies, Santa Clara, CA). The column temperature started at 120 °C for 2 min, increased at 25 °C/min to 200 °C, held for 2 min, ramped at 100 °C/min up to 280 °C, and then held 3.6 min at a helium carrier gas flow rate of 1.0 mL/min. The temperatures for inlet, ion source, and MS quadrupole were 250, 230, and 150 °C, respectively. The solvent delay and selected ion monitoring dwell time were set to 2 min. The selected ions for quantitation were *m*/*z* 68 and 93 for limonene and *m*/*z* 172 for the IS at a helium column flow rate of (2.5 ± 0.1) mL/min. Standard aqueous solutions of 1.00 mL in volume were prepared with limonene concentrations of 0.0, 0.1, 0.3, 0.5, 1, 5, 10, and 100 ng/μL in the presence of 1.0 μg/μL of the IS before the 2.5 μL injections. The areas under the appropriate peak for the analyte and IS were measured by using RTE manual integration. The area ratios of the limonene over the IS for each standard were plotted versus the corresponding standard solution concentrations or mass injected.

A cumulated permeated mass/area versus time plot for each permeation cell and hand experiment allowed the designation of SBT (time at the permeation rate of 100 ng/cm^2^/min) and calculation of lag time *t*_l_ (by extrapolation to the time axis of each linear steady state permeation period of the individual cumulated mass versus time plots). The diffusion coefficient was calculated from *D* = *L*^2^/6*t*_l_ where *L* is the original conditioned glove thickness.^15^ Averages and their standard deviations for all triplicate data were calculated and are listed in Table 1.

**Table 1:** Average Permeation Parameters at 35 °C for Conditioned Disposable Nitrile Gloves Relative to the Test Method (ASTM: ASTM F739-12 Permeation Cell in Moving Tray Water Bath; Robot NC, Robot Hand Non-clenching; Robot CL, Robot Hand Clenching) for Three Replicates for Each Glove Type^a^

**method**	**glove**	**A**	**B**	**C**	**D**	**E**	**F**
ASTM	Blue	128(3)	70(10)	13(2)	1.4(0.2	12(1)	3.04(0.08)
	Purple	104(2)	30(10)	69(6)	7.2(2.3)	17.2(0.7)	2.97(0.04)
	Sterling	73(2)	15(5)	77(10)	4.3(1.1)	17.1(0.8)	5.12(0.03)
	Lavender	59(3)	5(5)	295(30)	13.5(2.8)	20(1)	nd
robot NC	Blue	122(3)	30(10)	10(1)	9.2(2.9)	12(1)	3.04(0.07)
	Purple	105(2)	15(5)	67(5)	11.1(2.4)	17.2(0.7)	2.97(0.04)
	Sterling	74(3)	15(5)	100(7)	6.3(1.1)	17.1(0.8)	5.12(0.03)
	Lavender	58(2)	5(5)	423(31)	18(12)	20(1)	nd
robot CL	Blue	121(3)	30(10)	12(1)	9.9(1.2)	12(1)	3.04(0.07)
	Purple	106(3)	15(5)	78(7)	7.9(2.2)	17.2(0.7)	2.97(0.04)
	Sterling	74(4)	15(5)	104(6)	10.4(2.7)	17.1(0.8)	5.12(0.03)
	Lavender	59(3)	5(5)	490(31)	21(10)	20(1)	nd

a The quantities in parentheses are standard deviations for *n* = 3. A, palm glove thickness before permeation in μm; B, standardized breakthrough time in minutes; C, steady state permeation rate in ng/cm^2^/min; D, diffusion coefficient in units of cm^2^/min × 10^–8^; E, challenge surface acrylonitrile content in %; F, whole glove porosity in m^2^/g. nd: no data.

### 2.6 Other Measurements

Acrylonitrile content *A* was obtained by infrared reflectance spectroscopy with a diamond cell (Avatar 360 Fourier Transform Infrared Spectrometer, Thermo Nicolet, Madison WI) at the analytical wavelength of 2237 ± 5 cm^–1^. Seventeen poly(acrylonitrile-*co*-butadiene) reference materials with *A* contents of 10, 19–22, 30–35, and 37–39% (w/w) were obtained from Sigma–Aldrich (St. Louis, MO). The average weight percentage of acrylonitrile measured in triplicate for each reference material was used to prepare a standard curve.^16^

Porosity *P* data were from Mathews and Que Hee.^10,11^ In brief, gloves were punched into 3.18 mm diameter pieces and degassed at 80 °C with a nitrogen flow for 24 h, and the porosity in m^2^/g measured with a Micromeritics Tristar II 3020 Surface Area and Porosity System at a liquid nitrogen temperature of −196 °C assuming a Brunauer–Emmett–Teller (BET) adsorption of nitrogen as the probe gas, as prescribed by Micromeritics.

Linear regression and multivariate analyses with descriptive correlation coefficient *r* and probability *p*-value calculations were performed with Excel software in Microsoft Office, 2007.

Statistical significance is defined as *p* ≤ 0.05.

## 3 Results

The arithmetic mean permeation kinetic and glove characteristics data and their associated standard deviations for triplicates for the three test situations (the moving tray water bath modified ASTM method and the two whole glove permeation conditions, clenching and nonclenching for the YDRH) are presented in Table 1.

### 3.1 Thickness

The thicknesses of the palm sections of the conditioned whole gloves and the pieces examined in the ASTM tests were statistically the same for each glove type before and after permeation. The diffusion process was therefore Fickian. Thus, the initial thickness was used for all correlation analyses, and the calculation for the diffusion coefficient was valid. The order of increasing thickness was Lavender < Sterling < Purple < Blue. Blue was about twice the thickness of Lavender.

### 3.2 Standardized Breakthrough Time and Steady-State Permeation Rate

For each of the three tests, SBT averages followed the order Blue > Purple ≥ Sterling > Lavender, with Purple > Sterling only for ASTM. For Sterling and Lavender, the SBT average data for clenching and not clenching YDRH states and for the ASTM cell were statistically the same. The ASTM SBT data for Blue was 2.3 times the YDRH data and for Purple, twice. The precision of the data became worse at SBTs < 10 min rendering detection of differences difficult for Lavender.

For each of the three tests, SSPR averages followed the order Blue < Purple ≤ Sterling < Lavender, with Purple = Sterling only for ASTM. SSPR for Blue and Purple for the three tests for each glove type did not differ statistically. The YDRH data for the clenching hand were different from those when nonclenching only for the Lavender glove. The nonclenching YDRH data for Sterling and Lavender gloves were 1.3 and 1.4 times the respective ASTM values compared with 1.4 and 1.7 times, respectively, for the clenching robot hand data. Most of the data had imprecisions lower than 10% but no more than 15% (ASTM Blue).

The thickest glove (Blue) had the longest SBT and lowest SSPR and the thinnest glove (Lavender) had the shortest SBT and highest SSPR in each of the three tests. For the YDRH data sets, the Purple and Sterling gloves had equivalent SBT but the SSPR did differ statistically with Sterling being higher by 1.3–1.5 times.

### 3.3 Diffusion Coefficient

The average *D* value for Lavender was statistically higher than for Blue, Purple, and Sterling only in ASTM testing. Blue was also lower than Purple and Sterling, with Purple and Sterling having no statistical difference. While the data for YDRH data sets did not differ for Blue, the ASTM data for that glove were 0.141 (clenching) to 0.152 (nonclenching) of YDRH values.

### 3.4 Acrylonitrile Content and Porosity Comparisons

Challenge surface/collection surface *A* contents in % were as follows: Blue 12 ± 1/12 ± 1; Purple 17.2 ± 0.7/12.1 ± 0.7; Sterling 17.1 ± 0.8/12 ± 1; Lavender 20 ± 1/20 ± 1. The challenge surface values were chosen for comparisons since they are the first line of resistance. The *A* contents for challenge and collection sides are equal for Blue and for Lavender, with Lavender having the highest *A* content, 1.7 times the *A* content of Blue.

The *A* for the Purple and Sterling gloves is equivalent, but the *P* of the Sterling glove was 1.72 times that of the Purple glove. The *P* is equivalent for the Blue and Purple gloves, but Purple had 1.43 times more *A* than did Blue. Blue had the lowest *A* and *P* contents, and Lavender had the highest *A*.

### 3.5 Simple One-on-One Correlation Analyses

When each kinetic parameter (SBT, SSPR, and *D*) was regressed linearly one-on-one with the independent variables (*L*, *A*, and *P*), the following linear relationships in Table 1 units were as follows:

For ASTM:
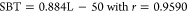
1
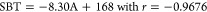
2
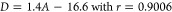
3
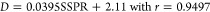
4
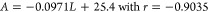
5

For the nonclenching robot hand:
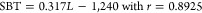
6
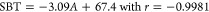
7

For the clenching robot hand:
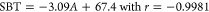
8
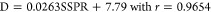
9

There are many more linear relationships for the ASTM data than for the robot hand. The SBT versus A inverse relationship was significant for all three sets of data. There were SBT identical relationships observed for the two YDRH conditions (eqs 7 and 8). The regression slope and intercept differed from those for the ASTM data.

The SBT versus *L* relationship was linear for the ASTM and the nonclenching robot hand, implying the two conditions were closer than that for the clenching robot hand.

*D* versus SSPR data were also linear for ASTM and the clenching robot hand but with different slopes and intercepts.

### 3.6 Multivariate Correlation Analyses

The preceding counterintuitive one-on-one correlation results suggested a multivariate approach might be more descriptive of form SBT α *L^x^Q A^y^P^z^* where *x*, *y*, and *z* are integers of either sign, and *Q* is the exposed permeation surface area. The inverse form is expected for SSPR. *D*, being a derived quantity from *t*_l_, was ignored as an independent variable. *Q* for the ASTM data is 5.06 ± 0.15 cm^2^, whereas *Q* for the robot hand is 1141 ± 73 cm^2^, 225 times more.^11^

The permeation procedures ensured that Q was constant for each permeation test type and not a factor, similar to temperature and preconditioning. The correlation coefficients *r* however can be compared across the same set of gloves although the regression equations cannot except for the two robot hand comparisons. The goal was to maximize *r*^*2*^ by varying *x*, *y*, and *z* as integers of either sign between 1 and 4 in an iterative manner. The most statistically significant relationships now follow:

For ASTM:
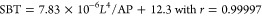
10
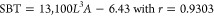
11
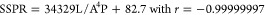
12

For the nonclenching robot hand:
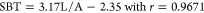
13
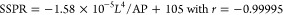
14

For the clenching robot hand:
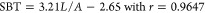
15
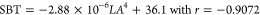
16
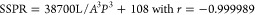
17

Only two relationships for SSPR were statistically significant for both clenching and nonclenching robot hand situations, those with *L*/*A*^2^*P* and *L*/*A*^3^*P*^4^. Neither was statistically significant for the ASTM data.

## 4 Discussion

### 4.1 Standardized Breakthrough Time

Accurate SBT values of less than 10 min were impossible to distinguish using a 10 min sampling protocol so that the clenching YDRH may have a SBT shorter than the nonclenching hand when both have SBTs < 10 min. This is likely for the Lavender glove but could not be demonstrated with the present data.

The one-on-one correlations revealed the expected direct linear relationship of SBT and *L* except for the clenching of YDRH. This implies that clenching perturbed the *L* dependence. In the study with cyclohexanol with the same dextrous robot hand system,^11^ only the nonclenching YDRH data also showed SBT versus *L* linearity.

The linearity of SBT with *A* had a negative *r* in the one-on-one analyses for all three permeation method sets (eqs 2, 7, and 8). The negative *r* was because of our infrared measurements for *A* were suggestive that the glove maker increased *A* for the thinner gloves to try to compensate for decreasing thickness (Table 1) and so is an artifact of glove design. The negative *r* is opposite to that expected since data in the literature support a direct linear relationship, that is, as *A* increases so too should SBT, other variables being constant but the thickness is not constant here.^16−20^ This indicates that one-on-one linear regression results are not necessarily cause and effect if interactions are occurring that may be better described through multivariate analysis.

The SBT results for the multivariate analyses also produced contradictory correlations. Equation 11 is preferred over eq 10 for ASTM since the slope and *r* are positive and SBT is directly related to both *L*^3^ and *A*. However, this was not observed for both robot hand situations.

### 4.2 Steady-State Permeation Rate

None of the limonene data showed one-to-one correlations of SSPR with *L*, *A*, or *P*. The only variable linearly related to SSPR for limonene was *D* for the ASTM and the clenching YDRH data. The relationships for *D* (eqs 3, 4 , and 9) have the expected direct linear dependence of *D* and SSPR but the direct relationship of *D* and *A* is counterintuitive. Multivariate regression analysis showed complex relationships that differed, all containing *L*, *A*, and *P* with *L* in the slope numerator and having a negative slope and *r* values. This does imply that *L* has the greatest direct influence but *A* and *P* cannot be ignored.

### 4.3 Related Matters

The SBT and SSPR data of the ASTM cell in the moving tray water bath at 35 °C predicted most permeation parameters of the YDRH, especially the SSPR of the two thickest gloves but less so for the observed SBT. The permeation testing data used for commercial permeation charts including disposable gloves based on ASTM F739 are collected near room temperature (20–27 °C), the 27 °C value being specified by ASTM F739–2020 unlike for previous ASTM F739 versions where no temperature was mandated.^3^ Such data will generally have longer SBT and lower SSPR than at 35 °C if permeation is not instantaneous at both temperatures. The Kimberly–Clark/Kimtech SBT and SSPR of limonene for the Sterling glove obtained with ASTM F739–1999 were^21^ 107 min and 0.157 pg/cm^2^/min, respectively, compared with average SBT of (15 ± 5) min and 77–104 ng/cm^2^/min for the average SSPR for the three permeation situations in the present study. It is to be noted that a vapor collection system was used by Kimberly–Clark/Kimtech for this chemical of high boiling point 175–176 °C.^22^ Here, the company SBT leads to an “Excellent” ranking (60–480 min) compared with “Good” (10–59 min) from the present results. There are no data available under the ASTM F739–2020 conditions.

Recent reviews^6,7^ have called for all permeation testing, but at least of disposable gloves, to be at 35 °C to match the ASTM-D6978 temperature.^5^ The results presented here also support this.

Another reason for the relative agreement of data from the ASTM F739 permeation cell in the moving tray water bath relative to the robot hand is that the forces produced by the solvent wave repetitive motion on the glove piece were designed to simulate gentle incident forces on the glove as well as mixing challenge and collection sides.^23^

The 1.8 kg of clenching force generated by the YDRH may also be too small to produce large differences in SBT and SSPR relative to no clenching. More clenches per unit time might also be effective but cause overheating so that a different dextrous robot hand would be necessary. Nevertheless, this 1.8 kg clenching force caused higher SSPR for the thinnest glove (Lavender) during limonene exposure in the present study relative to nonclenching, and similarly for the thinnest glove (Sterling) for the previous cyclohexanol challenge.^11^ This is indicative of an additional parameter to be reckoned with the glove-chemical interaction. The usual interaction term surrogate is the log_10_octanol/water coefficient (log_10_*K*_ow_) at 25 °C. The values for cyclohexanol and limonene are 1.23 and 4.57, respectively,^24^ that imply the polarity of a challenge chemical is important. More chemical challenges at 35 °C are necessary. The results do indicate that the whole glove dextrous robot hand test will be useful for thin materials.

Practically, the permeation breakthrough times of chemicals through disposable glove materials obtained at and about room temperature using ASTM F739 will generally be longer than at 35 °C and the SSPR smaller, providing a false sense of security and safety. If there is a choice of gloves, the one that is the most resistant relative to the expected exposure time should be chosen. If there is no choice or the chemical is unknown and there is no appropriate CPC available, double gloving should be adopted, this being shown to provide more resistance,^25^ however at the expense of hand dexterity in some cases. Some protection is better than no protection when no CPC is available.

## 5 Conclusions

Only the thinnest glove, Lavender, showed increased SSPR for the clenching dextrous robot hand relative to nonclenching on limonene challenge at 35 °C. The expected faster SBT for the clenching glove could not be observed because its SBT was less than the sampling period for both clenching and nonclenching states. Clenching did not affect the SBT and SSPR for the thicker gloves generally relative to ASTM F739 data at 35 °C. All permeations for each glove type resulted in the thickest glove, Blue, being the most resistant and the thinnest, Lavender, being the least. The multivariate analysis results were contradictory, but nitrile content and porosity do also influence SSPR. More studies with different chemicals and greater clenching forces are still needed to assess if a whole glove dextrous robot hand permeation standard is needed for the thinnest gloves.
